# Non‐compliant neutron dose associated with an internal lead slab in a radiotherapy treatment vault

**DOI:** 10.1002/acm2.70182

**Published:** 2025-07-26

**Authors:** Eduardo Galiano, Camila Salata, Marcelo Godin

**Affiliations:** ^1^ Autoridad Reguladora Radiologica y Nuclear ARRN Asuncion Paraguay; ^2^ Comissao Nacional de Energia Nuclear CNEN Rio de Janeiro Brasil; ^3^ Instituto Nacional del Cancer INCAN Asuncion Paraguay

**Keywords:** concrete, lead, neutron dose, photoneutrons, PRESCILA detector, radiotherapy, shielding, treatment vault

## Abstract

**Background:**

In high energy radiotherapy treatment vaults, photoneutrons can be produced by direct photon interactions with barrier materials and have a mean energy of approximately 20% of the nominal photon energy. Excess photoneutrons outside a treatment vault are always a concern due to their significant radiobiological efficacy, or Q value.

**Methods:**

A pre‐clinical regulatory inspection of a newly installed linear accelerator detected legally non‐compliant photoneutron dose levels in a controlled area outside the treatment vault. The non‐compliant dose was measured for a 10 MV photon beam external to the primary barrier improperly containing an internal 2.0 cm lead slab.

**Results:**

Based on measurements at a photon energy of 10 MV, using a workload *W* = 1500 Gy/week, a use factor of *U* = 1, and an occupancy factor of *T *= 0.5—which are NCRP‐151 recommended values—an equivalent neutron weekly dose of 443 ± 38 µSv was calculated for a point in a controlled area external to the vault (point A in Figure 1). This results in a calculated annual neutron dose at this point of 22.1 ± 2.2 mSv, which exceeds the legal occupational annual limit of 20 mSv for a controlled area in our jurisdiction. The lead slab was removed. Neutron measurements taken subsequent to the removal of the slab detected values slightly above natural background.

**Conclusions:**

The probable source of the non‐compliant neutron dose outside the treatment vault was the presence of the lead slab.

## 1 INTRODUCTION

In a radiotherapy treatment vault, photoneutrons produced by direct photon interactions with barrier materials have a mean energy of approximately 20% of the nominal photon energy.^1^ They can also be produced by particle evaporation, in which case the energy distribution is Maxwellian and unrelated to the incident photon energy. Photoneutrons are always a concern—particularly outside a treatment vault—due to their significant radiobiological efficacy, or Q value. The threshold energy for photonuclear reactions decreases roughly with increasing atomic number of the barrier material, which may result in unacceptable neutron doses outside the treatment vault. For example, the thresholds for lead and tungsten—two commonly used metals in accelerator treatment head components and/or as shielding materials—are 6.8 and 6.0 MeV, respectively.^1^ Few references were found in the literature regarding photoneutron measurements outside the primary barriers of a treatment vault for photon beams at or below 10 MV. This dearth of reports likely results from a consensus in the guiding literature, which either downplays—or dismisses—photoneutron production at or below 10 MV.^2^, ^3^, ^4^ This paradigm is now being challenged; for example, Rijken and collaborators have recommended that document NCRP‐151 be “updated” to incorporate more conservative concrete Tenth Value Layers (TVL's) for photon energies of 10 MV and below to more properly account for photoneutron production at these lower photon energies.^5^ One of the handful of references to actual physical measurements of neutron fields outside a treatment vault is the report by Carlone et al.^6^ These investigators reported that measured photoneutron dose rates for an 8 MV photon beam were approximately 1/10 of those at 10 MV. Swanson had previously reported photoneutron dose levels—external to a treatment vault—for photon energies as low as 5 MV!^1^ In a very specialized application, Jaradat and Biggs reported on the photoneutron contamination from an intraoperative unit operating at 9 MV.^7^ Chen et al. measured the neutron fields in and around a treatment vault generated by 10 MV photon beams using He‐3 proportional counters and bubble detectors.^8^ They concluded that for medical electron accelerators operating at photon energies of 10 MV or below, the neutron contamination may cause radiation safety problems if there is improper neutron shielding in place. Facure and collaborators simulated the effects of laminating the barriers of a treatment vault for a 10 MV photon beam, using the MCNP5 code.^9^, ^10^ They reported that laminating with steel did not present a problem external to the vault due to the higher photonuclear threshold energy of steel (∼13 MV); lamination with lead, however, proved to be a different story. For lamination with lead, the authors reported photoneutron dose equivalents outside the vault as high as 1.56 µSv per Gy of photon dose at isocenter. These dose levels integrated over 1 year can easily exceed the maximum legally permitted annual dose to occupational workers in many jurisdictions, including, for example, our own. More recently, Martin and McGuinley have also addressed in extenso, the issue of photoneutron yields in mixed metal/concrete barriers.^11^ In this work, we report on abnormally elevated neutron readings outside a treatment vault. The readings were likely associated with an incorrectly placed lead slab within one of the vault walls. As best we know, this may be the first such report.

## 2 METHODS

**FIGURE 1:**
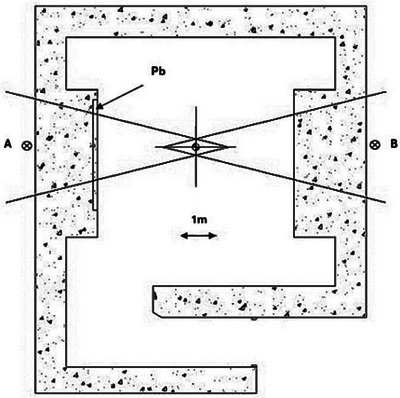
Treatment vault layout. The rectangular structure in the internal wall of the primary barrier to the left represents the lead slab. The center of the cross represents the treatment isocenter. Point A represents the point at which the non‐compliant neutron dose was measured.

**FIGURE 2:**
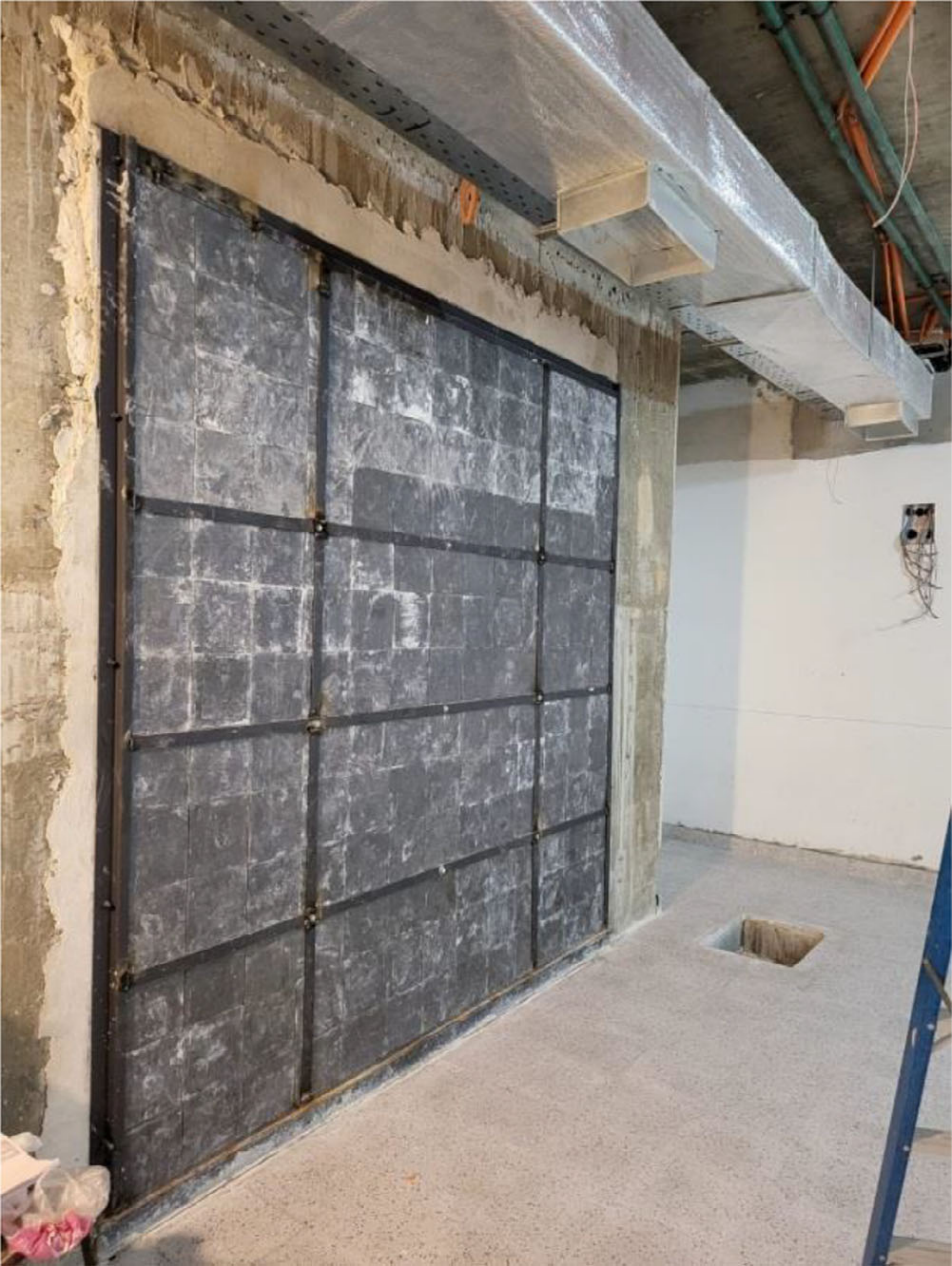
The 2.0 cm lead slab being installed on the inner wall of the primary barrier.

A post‐commissioning, pre‐clinical, external radiation survey of the treatment vault of a recently installed linear accelerator was undertaken by the National Regulatory Authority of Paraguay (ARRN) at the Instituto Nacional del Cancer (INCAN) Hospital in Paraguay (Varian Vital Beam II, Siemens Healthineers AG, Frankfurt, Germany). This is a 200 bed tertiary care facility that serves as the de‐facto national reference center for neoplastic diseases. The treatment vault in question was built during the Covid‐19 pandemic. For reasons that are not clear—but point to human error and lack of proper oversight—a vertical slab of lead bricks with a thickness of 2.0 cm was improperly placed in the internal wall of one of the primary barriers. The root cause of the improper placement of the slab is under investigation. The location of the slab inside the primary barrier is illustrated in Figure 1. Between the isocenter and point A, there are 190.0 cm of concrete and 2.0 cm of lead. Between the isocenter and point B, there are 220.0 cm of concrete only. A photograph of the placement of the lead slab during vault construction is presented in Figure 2. Due to anomalous neutron readings at point A, which were very likely associated with the presence of the lead slab, removal of the slab was undertaken, and the regulatory survey was then repeated.

All neutron measurements were made at a nominal photon energy of 10 MV with a 40 × 40 cm^2^ field size (at isocenter), at a gantry angle of 90° for point A and 270° for point B, at a dose rate of 600 MU/min. Based on historic data from an identical unit that has been operating at our institution for several years, we assign a *W* = 1500 Gy/week, with *U* = 1 and *T = *0.5, which are NCRP‐151 recommended values. The older unit has been treating an average of 100 patients/day with a mean dose of 3.0 Gy/fraction during working hours that go from 6:00 AM to 10:00 PM in two daily shifts. These parameters resulted in a measured equivalent neutron weekly dose of 443 ± 38 µSv at point A in Figure 1. The uncertainty is based on the uncertainty quoted by the primary laboratory for the calibration factor of the detector.

Neutron measurements were made with a Ludlum Model 2363 survey meter with a Proton Recoil Scintillator—Los Alamos (PRESCILA) Model 42‐41L neutron detector (Sweetwater, Texas, USA). The instrument was most recently calibrated on May 8th, 2023, at the Brazilian National Ionizing Radiation Metrology Laboratory (LNMRI/IRD). It was issued a 2 year calibration certificate; thus, all measurements were made within the period of validity of the calibration certificate. Conventional neutron rem meters rely on large moderator assemblies surrounding a thermal detector to achieve a rem‐like response function over a limited energy range. These typically present an ergonomic challenge, being heavy and bulky. The PRESCILA was developed as a low‐weight (2 kg) alternative capable of extended energy response, high sensitivity, and adequate gamma rejection. An array of ZnS(Ag) based scintillators is located inside and around a Lucite light guide, which couples the scintillation light to a photomultiplier tube. The use of both fast and thermal scintillators allows the energy response function to be optimized for a wide range of operational spectra. The inherent pulse height advantage of proton recoils over electron tracks in the phosphor grains eliminates the need for pulse shape discrimination and makes it possible to use the PRESCILA probe with standard pulse height discrimination provided by off‐the‐shelf health physics counters.^12^

Photon and neutron measurements were made at external points to the vault, consistent with recommendations in chapter six of NCRP‐151.^4^ In this report, we only address neutron measurements, as all photon measurements were legally compliant with regulatory requirements. Primary barrier transmission measurements were made without a phantom. The mean weekly dose *D* (in µSv/week) is given by the following expression:

(1) $$D=\left(L-L_{f}\right)*T*U*W/\left(60*D_{\mathrm{nom}}\right)$$

where *L* and *L_f_* are the actual and background readings, both in µSv/h, *T* is the occupancy factor, *U* is the use factor, *W* is the workload at isocenter in Gy/week, and *D*_nom_ is the maximum dose rate at isocenter in Gy/min. The *U* and *T* factors used were those recommended in NCRP‐151.^4^

## 3 RESULTS AND DISCUSSION

The calculated annual neutron dose to point A was 22.1 ± 2.2 mSv, which exceeds the legal occupational annual limit of 20 mSv for a controlled area in our jurisdiction. For comparison, the calculated annual photon dose to point A was 2.39 ± 0.24 mSv—comfortably within regulatory requirements. The vault was therefore deemed by the ARRN to be legally non‐compliant with existing regulations, and an operating certificate for 10 MV photons was not issued to the INCAN Hospital, pending remedial action.

No neutron dose above background was measured at external point B behind the contralateral primary barrier, which lacked a lead slab. A working group (the authors of this report) consisting of the head of medical physics at INCAN Hospital (MG), a radiological physicist from the ARRN (EG), and an external expert sent by the International Atomic Energy Agency (CS) quickly came to the unanimous conclusion that the source of excess photoneutrons was most likely the lead slab improperly placed in the primary barrier. Based on this consideration, the recommendation for remedial action was the removal of the lead slab, which the hospital administration acted on. There was sufficient concrete thickness in the primary barrier to properly shield 10 MV photons as per the NCRP‐151 methodology, so photon shielding was never a regulatory concern. A thorough post lead‐slab‐removal external survey revealed neutron dose levels slightly above natural background at point A and photon dose levels statistically unchanged compared to pre slab‐removal values. An interesting potential extension of this work—beyond the scope of this brief communication—would involve the stochastic modelling of neutron self‐shielding in the lead slab and concrete wall, using, for example, the MCNP code.^10^, ^13^

## 4 CONCLUSIONS

Upon a post‐commissioning, pre‐clinical, regulatory inspection of a recently installed medical linear accelerator, a legally non‐compliant photoneutron dose level was measured in a controlled area outside the vault for a 10 MV photon beam. The offending excess dose was measured external to a primary barrier improperly containing an internal 2.0 cm lead slab. Measurements external to the otherwise similar contralateral primary barrier, which is devoid of lead, revealed neutron doses indistinguishable from background. After removal of the lead slab, measured neutron dose levels external to the vault were slightly above background. It was therefore concluded that a probable source of the excess photoneutrons was the lead slab.

## AUTHOR CONTRIBUTIONS

C. Salata and M. Godin aquired, anlyzed, and reduced data. E. Galiano prepared the manuscript.

## CONFLICTS OF INTERESTS STATEMENT

The authors report no conflicts of interest.
